# The Role of Fecal Calprotectin in Evaluating Intestinal Involvement of Behçet's Disease

**DOI:** 10.1155/2016/5423043

**Published:** 2016-08-24

**Authors:** Burak Özşeker, Cem Şahin, Havva Solak Özşeker, S. Cumali Efe, Taylan Kav, Yusuf Bayraktar

**Affiliations:** ^1^Division of Gastroenterology, Department of Internal Medicine, School of Medicine, Muğla Sıtkı Koçman University, 48000 Muğla, Turkey; ^2^Department of Internal Medicine, School of Medicine, Muğla Sıtkı Koçman University, 48000 Muğla, Turkey; ^3^Department of Pathology, Education and Research Hospital, Muğla Sıtkı Koçman University, 48000 Muğla, Turkey; ^4^Gastroenterology Unit, Batman State Hospital, 72070 Batman, Turkey; ^5^Department of Internal Medicine, Division of Gastroenterology, School of Medicine, Hacettepe University, 06230 Ankara, Turkey

## Abstract

One of the regions of involvement of Behçet's disease (BD), a systematic inflammatory vasculitis with unknown etiology, is the gastrointestinal (GI) tract. Upper GI endoscopy, colonoscopy, and capsule endoscopy are frequently used methods to diagnose the intestinal involvement of BD. The aim of this study was to investigate the role of fecal calprotectin (FC) in the evaluation of intestinal involvement in BD.* Material and Method*. A total of 30 patients who were diagnosed with BD and had no GI symptoms and 25 individuals in the control group were included in this study.* Results*. Levels of FC were statistically significantly higher in patients with BD compared to the control group (*p* < 0.001). The correlation analysis performed including FC and markers of disease activity revealed a positive and statistically significant correlation between FC level and CRP and erythrocyte sedimentation rate (*r*: 0.255, *p* < 0.049, and *r*: 0.404, *p* < 0.001, resp.). FC levels in patients who were detected to have ulcers in the terminal ileum and colon in the colonoscopic examination were statistically significantly higher compared to the patients with BD without intestinal involvement (*p* = 0.01).* Conclusion*. The measurement of FC levels, in patients with BD who are asymptomatic for GI involvement, may be helpful to detect the possible underlying intestinal involvement.

## 1. Introduction

Behçet's disease (BD) is a systemic inflammatory vasculitis with unknown etiology and manifests with recurrent oral aphthous ulcers, genital ulcers, skin lesions, and eye involvement and the involvement of the vascular, neurological, and gastrointestinal (GI) systems [1]. According to the International Study Group criteria, the diagnosis of BD can be made in the presence of two of genital ulcers, typical ophthalmic findings, typical skin lesions, or positive pathergy test in addition to oral aphthous ulcers [2].

One of the regions of involvement in BD is GI involvement and the disease is then called intestinal BD or enterobehçet disease. The most frequent involvement of BD in the gastrointestinal system is observed as recurrent oral aphthous ulcers, which are frequently the first symptom of the disease in almost all patients and stand for one of the diagnostic criteria. Nevertheless, involvement of esophagus, stomach, duodenum, and small and large bowel is also seen in GI involvement. Symptomatic involvement of the esophagus is extremely rare and commonly accompanies other GI manifestations [3]. The least frequently affected organ is the stomach in the GI system and the most common finding is aphthous gastritis or duodenal ulcers when the involvement is present.

Involvement of the small and large intestines can be evaluated under two groups, such as luminal and vascular involvement. Ulcers can develop secondary to small vessel involvement and mucosal inflammation and intestinal infarcts can be seen secondary to large vessel involvement. Luminal involvement starts from the oral mucosa and manifests generally as ulcers in the esophagus, stomach, and intestines in varying degrees, while vascular involvement is characterized as aneurysms or thrombosis in the large vessels in general. Mucosal ulcerations are most frequently seen in the ileocecal region [4]. Since ulcers penetrate all the colonic walls, perforations from multiple sites, fistula formation, or bleeding can develop frequently [5].

According to the current knowledge on the disease, there is no specific marker for the intestinal involvement of BD. Fecal calprotectin (FC) is a cytosolic protein, generally present in the neutrophils and macrophages, and exerts an antimicrobial effect by binding to calcium. FC becomes evident during cell activation or death. We encountered a very low number of studies investigating the level of FC in BD in the literature review we performed [6]. The aim of this study was to investigate the role of FC in the evaluation of intestinal involvement in BD.

## 2. Material and Method

### 2.1. Patient Selection and Evaluation

This study was performed in the Gastroenterology Unit of the Department of Internal Medicine, School of Medicine at Hacettepe University, after the approval of the local ethics committee. The patients included in the study were composed of individuals who were older than 18 years and were diagnosed with BD according to the International Study Group criteria. Patients with BD who presented to the gastroenterology and rheumatology outpatient clinics between January 1, 2012, and April 30, 2012, were evaluated to be included in the study and patients who consented to participate in the study were evaluated for suitability to the study criteria. All patients with BD were questioned for GI symptoms in detail. Patients without any GI symptoms were included in the study. Upper endoscopy and colonoscopy were performed in patients to evaluate the GI involvement. Test results (pathological examination and rapid urease test) for* H. pylori* during upper GI interventions were recorded. In colonoscopic examinations, findings including the terminal ileal region and pathological examination results were recorded.

Cases that were detected to have mucosal ulceration in the terminal ileum and/or proximal colonic segments during colonoscopy examination were accepted as positive for intestinal involvement. The control group is composed of patients matched for age and gender among those presenting to the outpatient clinics during the same period stated above.

### 2.2. Laboratory Examinations

Venous blood samples (20–25 mL) were drawn following an overnight fasting of 8–12 hours between 08:00 and 09:00 am. Some 2.5 mL of the collected blood was transferred to a tube containing ethylenediaminetetraacetic acid (EDTA) and whole blood count and erythrocyte sedimentation rate (ESR) counting were performed in one hour. The rest of the blood was transferred to two normal tubes and the tubes were left for 10 minutes until clotting occurred. The serum obtained following centrifugation at 4000 rpm at room temperature was transferred to the Eppendorf tubes and was stored at −40°C to be analyzed at the end of the study. Patients were asked to give stool samples for FC detection on the same day the blood samples were obtained and stool samples were analyzed on the day they were obtained.

CRP level was measured using “IMMAGE 800 immunochemistry system” equipment (Beckman Coulter Inc., Ireland) with the nephelometric method and original kits.

“*BÜHLMANN Quantum Blue Reader*” equipment was used in the FC analysis. Samples arriving at the laboratory were analyzed on the same day. Stool samples were prepared following extraction after diluting to 1/15 using an “extraction buffer.” The mixture obtained was mixed well and the diluted extracts were centrifuged at 3000 rpm for 5 minutes. At the last stage, a FC cartridge was prepared using the Lateral Flow Assay method. Some 60 *μ*L of diluted and centrifuged stool extract was transferred to the test mixture. The device was started. The device read the test cartridge automatically after 12 minutes and it calculated the result. The test results were confirmed. The results of the samples with more than 300 *μ*g/g were further diluted and retested. Ultimate result was calculated by multiplication of the obtained result by the dilution factor.

### 2.3. Statistical Analysis

Data was analyzed using SPSS software version 20.0 for Windows (SPSS Inc., Chicago, Illinois, USA). The distribution of continuous variables was performed using Kolmogorov-Smirnov test and homogeneity test. Numerical variables without a normal distribution were expressed as median (min-max), while normally distributed numerical variables were expressed as mean (±sd). Logarithmic conversion was applied to the numerical variables with nonnormal distribution. The Mann-Whitney *U* test was used in the comparison of means of the non-normally distributed quantitative variables following logarithmic conversion, while the independent-samples *t*-test was used in the comparison of means of normally distributed quantitative variables. The chi-square test was used in the analysis of categorical variables. A ROC curve analysis was performed to define a cutoff value for FC level as a marker for the intestinal involvement of BD. As a result of the ROC curve analysis, the FC level with a highest likelihood ratio (LR) value was defined as the cutoff value. The statistical significance of the data obtained was interpreted using a “*p*” value; *p* < 0.05 was accepted as statistically significant.

## 3. Results

Thirty patients with BD and 25 healthy volunteers as control group were included in the study. No statistically significant difference was found in age and gender between the patient and control groups. Demographic data of the patients are summarized in Table 1.

When the markers of disease activity between the patient and control groups were compared, ESR and FC levels were statistically significantly higher in patients with BD group compared to the control group (*p* < 0.001 and *p* < 0.001, resp.). However, no statistically significant difference was found in the CRP levels between the patients with BD and control group (*p* = 0.235). The correlation analysis between FC level and markers of disease activity demonstrated a positive and statistically significant correlation between CRP and ESR levels and FC level (*r*: 0.255, *p* < 0.049, and *r*: 0.404, *p* < 0.001, resp.).

In this study, colonoscopy and upper GI endoscopy were performed in 30 patients who were diagnosed as BD and were asymptomatic for GI symptoms. As a result of those examinations, no pathology was detected in 24 patients by colonoscopy and upper endoscopy, while one patient was diagnosed to have mucosal edema, granularity, and fragility, compatible with terminal ileitis, while superficial mucosal ulcers with a diameter varying between 0.5 and 1 cm were detected in five patients in the terminal ileum. Other causes of terminal ileitis, including Crohn's disease, were excluded by clinical and histological examinations. In the five patients with ulcers in the terminal ileum, larger ulcer size was not associated with higher levels of FC (*p* = 0.23).

Patients with BD were divided into two groups according to positive (*n*: 6, 20%) and negative (*n*: 24, 80%) intestinal involvement and markers for disease activity in these groups were demonstrated in Table 2. No statistically significant difference was found in CRP and ESR between the groups, while FC levels were statistically significantly higher in the group with positive intestinal involvement (*p* = 0.010).

**Figure 1:**
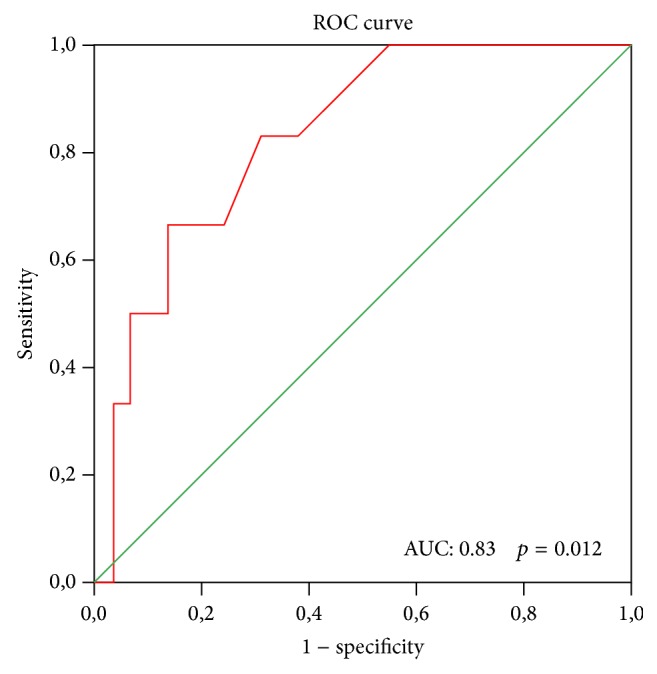
ROC curve analysis of FC level to predict intestinal involvement in Behçet's disease.

ROC curve that was performed to define a cutoff value for FC as a marker of intestinal involvement in the BD group revealed a cutoff value for FC of 49.5 *μ*g/g with 83.3% sensitivity and 69% specificity (LR = 2.68) (*p* = 0.012, AUC: 0.83) (Figure 1).

## 4. Discussion

FC levels were statistically significantly higher in cases with BD compared to the control group in this present study. In addition, no statistically significant difference was found in the levels of classical inflammatory markers such as CRP and ESR between patients with BD with positive and negative intestinal involvement, while FC levels were statistically significantly higher in the group with positive intestinal involvement and the group with negative intestinal involvement.

Gastrointestinal system involvement of BD has been known to occur more frequently in Far East countries [7]. The incidence of enterobehçet disease in Turkey has been reported to be 1.4% in a previous study [8]. In circumstances when complaints related to intestinal involvement of BD are the initial symptoms or dominant symptoms, patients might be misdiagnosed as inflammatory bowel disease or other pathologies [7]. Therefore, other diseases such as Crohn's disease, tuberculosis, and vasculitis should be excluded before the diagnosis of intestinal involvement of BD is made [9].

Methods such as upper GI endoscopy, colonoscopy, and capsule endoscopy are frequently used in the diagnosis of the intestinal involvement of BD. Therefore, colonoscopy must be performed in cases with clinical symptoms. The most common colonoscopic finding is single or multiple ulcers localized in the ileocecal region [10]. A large ulcer with a typical ovoid form or ulcerations in the small or large intestine in a patient matching the diagnostic criteria of the BD leads to the diagnosis of intestinal involvement of the BD. Nevertheless, capsule endoscopy is recommended in cases with BD with gastrointestinal system complaints where no pathology is detected, especially in endoscopy, colonoscopy, and upper gastrointestinal series with barium [11, 12]. Current knowledge indicates no specific marker associated with the intestinal involvement of BD. However, various markers have been investigated to evaluate the intestinal involvement. One of those markers is FC that has been used to diagnose inflammatory bowel diseases (IBD) in particular.

Fecal calprotectin is a cytosolic protein that generally is present in neutrophils and macrophages and is liberated during cell activation and death. It binds to calcium and exerts an antimicrobial effect. Calprotectin, called L1 protein when first isolated from granulocytes in 1980, has been given the current name after its binding to calcium and antimicrobial effect has been defined. Calprotectin constitutes 50–60% of the neutrophil cytosol proteins and it is liberated during cell activation or death. It may stay stable for approximately seven days following elimination in the stool. Its detection in the stool is performed by the “enzyme-linked immunosorbent assay” (ELISA) method.

Although FC is not used currently in the routine clinical practice, it is sometimes used in the diagnosis of some diseases such as inflammatory bowel disease (IBD). In a meta-analysis in which FC detection was evaluated in IBD, 13 prospective studies were analyzed including six studies in adults and seven studies in children and increased presence of FC was demonstrated to be 93% sensitive and 96% specific in the diagnosis of IBD [13]. In addition, it was established that the detection of FC as a screening test might decrease the rate of possible unnecessary endoscopic examinations. Wright demonstrated that FC levels can help to distinguish between the patients with positive symptoms who will benefit from colonoscopy and whose colonoscopy would be normal [14].

Mucosal healing is important criteria of remission in IBD. Colonoscopic examinations may not always reveal definite mucosal healing as microscopic inflammation can continue. In a recently published study, Theede et al. demonstrated that FC levels may predict histological mucosal healing in ulcerative colitis patients [15].

The quantitative measurement of FC was used as a screening test in a recent study, which is recommending colonoscopy for patients with FC levels higher than 150 *μ*g/g for possible organic diseases such as IBD and colorectal cancer. It is cited that, for the patients with FC levels between 50 and 150 *μ*g/g, the bowel inflammation is likely. In our study, we had a median FC level of 48 (33–770) and 18 (8–30) for patients with entero-BD and BD patients without GI involvement, respectively. The results of our study and studies reveal that FC levels can distinguish between the patients who need colonoscopic examination in IBD and BD [16].

Based on its nature as a marker demonstrating an increased presence of activated neutrophils in the intestinal lumen, an increased FC level has been established to be useful in patients with a preliminary diagnosis of IBD and its use as a screening test has been established to decrease the possibility of negative endoscopy in some studies. However, since FC levels were increased in BD, in addition to some inflammatory, infectious, and malignant conditions, it has been discouraged to be used as a screening test for IBD as was mentioned in the same direction in some guidelines [13].

In a recent study, FC levels of 51 patients with Ankylosing Spondylitis (AS) were compared with control group and FC levels were found to be statistically significantly higher in AS patients, compared to the control group [17].

No study was encountered during the literature review evaluating the FC level in BD. In a study in which serum calprotectin level was analyzed in BD, calprotectin serum levels were found to be significantly higher in cases with BD compared to healthy controls; however serum calprotectin levels demonstrated no correlation with BD activity in that study [6].

In the present study, we demonstrated presence of intestinal involvement as ileitis and ulcers in terminal ileum in six patients with BD, which were completely asymptomatic. No significant differences were observed between cases with BD positive and negative for intestinal involvement in the levels of classical inflammatory markers such as CRP and ESR, while FC levels were statistically significantly higher in the group positive for intestinal involvement compared to the group with negative intestinal involvement. Also, in the present study, the cutoff value for FC was 49.5 with 83.3% sensitivity and 69% specificity as a marker for intestinal involvement (*p* = 0.012).

Our study has some limitations. The number of patients enrolled in this study is low for a definitive statement about the role of FC in entero-BD patients. Also, we did not compare the sensitivity and specificity of FC with other IBD diseases, as we did not enroll any patients with IBD for this study.

In light of this study, it is suggested that FC measurement may be a guide in clinical evaluation of possible underlying GI involvement in cases with asymptomatic BD provided that other inflammatory, infectious, and malignant conditions that might increase the level of FC are excluded. Further multicentered prospective studies are needed in order to prove our results.

## Figures and Tables

**Table 1:** Demographic and laboratory values of patients included in this study.

Variable	Behçet's disease	Control	*p* value
Number of patients	30	25	
Age, years	40.29 ± 11.50	37 ± 5.66	0.179
Gender (M/F)	16/19	14/11	0.441
Hb, g/dL	13.71 ± 1.66	14.58 ± 1.43	0.065
MCV, fL	84.54 ± 5.60	87.22 ± 4.18	**0.041**
WBC, (mm^3^)	7120 ± 1895	6816 ± 1578	0.589
^*∗*^Platelets, 10^3^ mm^3^	228 (116–532)	224 (144–361)	0.509
^*∗*^ALT, U/L	19 (8–132)	16 (10–40)	0.221
^*∗*^AST, U/L	20 (11–91)	18 (10–41)	0.223
^*∗*^ALP, U/L	73 (17–159)	63 (45–102)	0.052
^*∗*^GGT, U/L	22 (4–100)	15 (10–37)	0.105
^*∗*^ESR, mm/hour	15 (2–64)	6 (2–12)	**<0.001**
^*∗*^CRP, mg/dL	0.69 (0.01–10.9)	0.04 (0.01–0.20)	0.235
^*∗*^Fecal calprotectin *μ*g/g	48 (33–770)	18 (8–30)	**<0.001**

Non-normally distributed parameters with “*∗*” sign were expressed as median (minimum–maximum).

WBC: white blood cell, Hb: hemoglobin, MCV: mean corpuscular volume, ALT: alanine aminotransferase, AST: aspartate aminotransferase, ALP: alkaline phosphatase, GGT: gamma-glutamyl transferase, ESR: erythrocyte sedimentation rate, and CRP: C-reactive protein.

**Table 2:** Comparison of inflammatory markers in the patient group according to intestinal involvement.

Variable	Patients with BD with GI involvement	BD without GI involvement	*p *value
WBC, (mm^3^)	7120 ± 1895	6800 ± 1578	0.146
^*∗*^ESR, mm/hour	15 (2–64)	6 (2–12)	0.696
^*∗*^CRP, mg/dL	0.387 (0.01–4.2)	0.21 (0.01–0.20)	0.190
^*∗*^Fecal calprotectin *μ*g/g	48 (33–770)	18 (8–30)	**0.010**

Non-normally distributed parameters with “*∗*” sign are expressed as median (minimum–maximum).

WBC: white blood cell, ESR: erythrocyte sedimentation rate, and CRP: C-reactive protein.
